# Crystal structure of CD27 in complex with a neutralizing noncompeting antibody

**DOI:** 10.1107/S2053230X17005957

**Published:** 2017-04-26

**Authors:** Alexey Teplyakov, Galina Obmolova, Thomas J. Malia, Gary L. Gilliland

**Affiliations:** a Janssen Research and Development LLC, 1400 McKean Road, Spring House, PA 19477, USA

**Keywords:** CD27, cysteine-rich domain, antibodies, crystal structure, epitopes, tumor necrosis factor

## Abstract

CD27 is a T-cell and B-cell co-stimulatory glycoprotein of the tumor necrosis factor (TNF) receptor superfamily that is dependent on the availability of the TNF-like ligand CD70. Therapeutic approaches to treating autoimmune diseases and cancers with antagonistic and agonistic anti-CD27 monoclonal antibodies (mAbs), respectively, have recently been developed. Mouse anti-human CD27 mAb 2177 shows potency in neutralizing CD70-induced signaling; however, it does not block the binding of soluble CD70. To provide insight into the mechanism of action of the mAb, the crystal structure of the CD27 extracellular domain in complex with the Fab fragment of mAb 2177 was determined at 1.8 Å resolution. CD27 exhibits the assembly of cysteine-rich domains characteristic of the TNF receptor superfamily. The structure reveals a unique binding site of mAb 2177 at the edge of the receptor molecule, which allows the mAb to sterically block the cell-bound form of CD70 from reaching CD27 while leaving the ligand epitope clear. This mode of action suggests a potential dual use of mAb 2177 either as an antagonist or as an agonist.

## Abstract 2

The crystal structure of human CD27 was determined at a resolution of 1.8 Å, revealing the assembly of the cysteine-rich domains. The binding of the antibody at the edge of the receptor molecule is unusual for the tumor necrosis factor receptor superfamily.

## 1. Introduction   

CD27 is a type I transmembrane glycoprotein expressed as a surface antigen on T cells, natural killer cells and antibody-secreting plasma and memory B cells (Borst *et al.*, 2005 ▸). CD27 is required for the generation and long-term maintenance of T-cell immunity. CD27 belongs to the tumor necrosis factor (TNF) receptor superfamily, which also includes OX40, CD40, CD30, receptors of nerve growth factor, lymphotoxin beta and Fas, and a number of death receptors (Camerini *et al.*, 1991 ▸). Similarly to the other members of the superfamily, the extracellular portion (ECD) of CD27 contains several cysteine-rich domains (CRD).

The only known ligand of CD27 is CD70, a trimeric type II transmembrane protein of the TNF ligand superfamily. CD70 expression is normally restricted to B cells, mature dendritic cells and activated T cells (Nolte *et al.*, 2009 ▸; Denoeud & Moser, 2011 ▸). CD27–CD70 ligation results in activation of NF-κB signaling pathways, which in turn stimulates B-cell and T-cell proliferation, plasma cell differentiation and subsequent antibody secretion (Yamamoto *et al.*, 1998 ▸). Studies in humans and animals suggest an important role of the CD27–CD70 pathway in various immune-related diseases, including systemic lupus erythematosus (Dörner & Lipsky, 2004 ▸), rheumatoid arthritis (Tak *et al.*, 1996 ▸) and multiple sclerosis (Hintzen *et al.*, 1991 ▸). CD27 may also control the accumulation of CD4^+^ and CD8^+^ T cells at sites of infection (Hendriks *et al.*, 2000 ▸). While agonist anti-CD27 antibodies may be useful for promoting T-cell-mediated antitumor or antiviral immunity (He *et al.*, 2013 ▸), antagonist antibodies can exert a clinically useful cytotoxic, cytostatic or immunomodulatory effect on CD27-expressing cells, particularly without causing undesirable agonist effects in the absence of CD70 (Makino *et al.*, 2012 ▸). The therapeutic targeting of the CD70–CD27 system has been thoroughly covered by a recent review (Wajant, 2016 ▸). However, a number of open questions regarding the structure–function relationship of agonistic and antagonistic antibodies and the importance of the epitope and affinity in their biological potency remain.

Anti-CD27 monoclonal antibody (mAb) 2177 was obtained from mouse hybridoma against human CD27 ECD-Fc fusion protein. MAb 2177 binds CD27 with a monovalent affinity in the low-nanomolar range and shows potency in neutralizing CD70-induced signaling (Obmolova *et al.*, 2017 ▸). Surprisingly, mAb 2177 does not prevent the binding of soluble CD70 (sCD70) to CD27, indicating that it does not compete for the same epitope (Obmolova *et al.*, 2017 ▸). The mAb shows no agonistic activity *in vitro* without additional cross-linking or *in vivo* when presented on a silent IgG isotype (Chen *et al.*, 2015 ▸).

To provide structural insight into the functional properties of the antibody, we determined the crystal structure of the ECD of human CD27 in complex with the Fab fragment of mAb 2177. Identification of the mAb epitope adds to the continued discussion on the structurally distinct epitopes in the TNF receptor (TNFR) superfamily and their possible relationship to different biological outcomes. This 1.8 Å resolution structure of CD27 in complex with Fab 2177 is more complete and more accurate than that in a ternary complex with Fabs 2177 and 2191 which was determined previously at 2.7 Å resolution (Obmolova *et al.*, 2017 ▸). It extends our knowledge of the TNFR superfamily and will be useful for studies of receptor–ligand and drug–target interactions. The structure also allowed a mapping of the pathological mutations in CD27 related to immunodeficiency.

## 2. Materials and methods   

### 2.1. Proteins   

The CD27 construct used for crystallization contained amino acids 1–101 of human CD27 (21–121 of the full-length sequence according to the UniProtKB entry CD27_HUMAN) with a 6×His tag at the C-terminus. The protein was expressed in baculovirus-infected Sf9 insect cells (*Spodoptera frugiperda*) and was purified at Proteos Inc. (Kalamazoo, Michigan, USA) using metal-ion chromatography on an Ni–NTA column (Thermo Fisher) and size-exclusion chromatography (SEC) on a Superdex 200 column (GE Healthcare). It was further purified in-house on a Mono S column (GE Healthcare). No deglycosylation was attempted.

The Fab fragment of mAb 2177 was constructed by fusing the mouse variable domains with human IgG1/κ constant domains that contained a 6×His tag at the C-terminus of the heavy chain. Two Lonza-based vectors (Lonza Group, Switzerland), p4275 and p4208, were used to construct expression plasmids for IgG1 heavy chain and κ light chain, respectively, following the protocol described previously (Zhao *et al.*, 2009 ▸). The Fab was expressed in HEK 293 cells and was purified by affinity and size-exclusion chromatography using HisTrap and Superdex 200 columns, respectively (GE Healthcare).

### 2.2. Crystallization   

The CD27–Fab 2177 complex was prepared by mixing the Fab with a 25% molar excess of CD27 in 20 m*M* Tris pH 8.5, 250 m*M* NaCl and incubating at 4°C overnight. Formation of the complex was monitored on a Superdex 200 column. The unbound fraction (CD27) showed as a very minor peak, and the complex was crystallized without further purification. The solution was concentrated to 12 mg ml^−1^ using an Amicon Ultra 10 kDa device.

Crystallization of the complex was carried out by the vapour-diffusion method at 20°C using an Oryx4 robot (Douglas Instruments). The experiments were composed of equal volumes of protein and reservoir solution in a sitting-drop format in 96-well Corning 3550 plates. Initial screening was performed with The PEGs Suite (Qiagen) and in-house screens (Obmolova *et al.*, 2010 ▸). A hit was observed in 25% PEG 3350, 0.1 *M* MES pH 6.5 and was used to prepare seeds for microseed matrix screening (D’Arcy *et al.*, 2007 ▸; Obmolova *et al.*, 2010 ▸). Crystals suitable for X-ray analysis were obtained from 17% PEG 4000, 0.2 *M* ammonium citrate in 0.1 *M* MES buffer pH 6.5.

### 2.3. X-ray data collection and structure determination   

For X-ray data collection, one crystal was soaked for a few seconds in a cryoprotectant solution containing mother liquor supplemented with 20% glycerol and flash-cooled in liquid nitrogen. X-ray diffraction data were collected at the Swiss Light Source using a PILATUS 6M detector. Diffraction intensities were detected over a 180° crystal rotation with 0.25 s exposures per 0.25° image. The data were processed with *XDS* (Kabsch, 2010 ▸). X-ray data statistics are given in Table 1 ▸.

The structure was determined by molecular replacement with *Phaser* (McCoy *et al.*, 2007 ▸) using the variable and constant domains of anti-Fas Fab (PDB entry 1iqw; Ito *et al.*, 2002 ▸) as search models. When the Fab was positioned in the unit cell, the CD27 molecule was manually traced in the electron density using *Coot* (Emsley *et al.*, 2010 ▸). All crystallographic calculations were performed with the *CCP*4 suite of programs (Winn *et al.*, 2011 ▸). Refinement statistics are given in Table 1 ▸. Ramachandran statistics were calculated with *PROCHECK* (Laskowski *et al.*, 1993 ▸). The solvent-accessible surface area and shape complementarity were calculated with *AREAIMOL* and *SC* from the *CCP*4 suite. Figures were prepared with *PyMOL* (Schrödinger).

## 3. Results and discussion   

### 3.1. CD27 structure   

The crystal structure of the CD27–Fab 2177 complex was determined at 1.8 Å resolution. The electron density is good everywhere except for the His tags at the C-termini of CD27 and the Fab heavy chain. The electron density suggests that there is one N-glycosylation site, at Asn75, where an *N*-acetylglucosamine moiety was included in the model. Compared with the CD27 structure in the ternary complex with Fab 2177 and Fab 2191 (PDB entry 5tlk; Obmolova *et al.*, 2017 ▸), no significant differences are observed. The r.m.s.d. for 75 common C^α^ atoms in the two structures is 0.86 Å.

CD27 has an elongated ladder-like structure composed of cysteine-rich domains (CRD), each of about 40 residues in length. Residues 1–43 form CRD1, residues 44–85 form CRD2 and residues 86–101 form CRD3 (the numbering of the mature form is used throughout the paper). While CRD1 and CRD2 contain three disulfides each, the third CRD is incomplete and contains only two disulfides: 86–100 and 92–97. (The UniProtKB annotation for entry CD27_HUMAN incorrectly assigns the disulfides as 86–97 and 92–100.)

**Figure 1:**
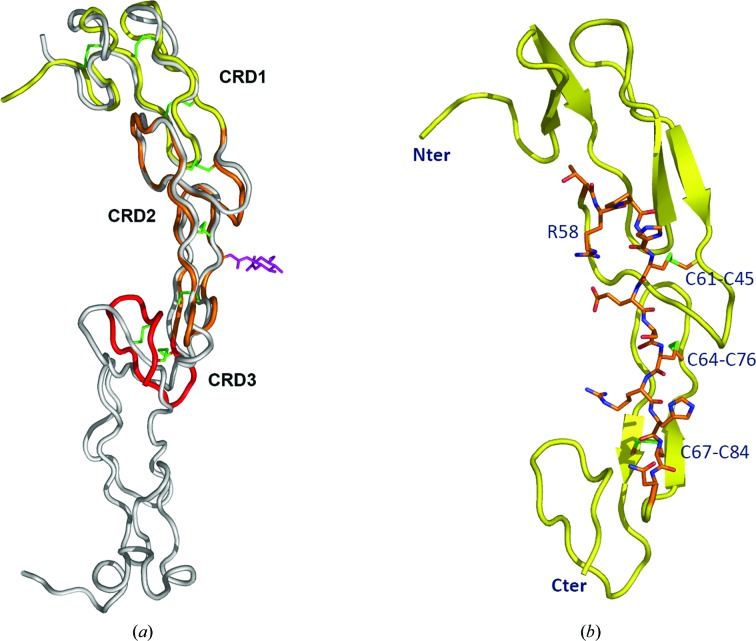
Structure of CD27. (*a*) Superposition of CD27 from the present structure on the TNFR1 structure (PDB entry 1ext). CRDs are shown in different colors and TNFR1 is in gray. Disulfides (green) and N-glycosylation at Asn75 (magenta) are shown as sticks. (*b*) CD27 ECD with the putative CD70 epitope (residues 57–68) shown as sticks. Mutation of Arg58 leads to immunodeficiency.

The overall fold and the disulfide pattern in CRD1 and CRD2 are typical of the TNFR superfamily (Banner *et al.*, 1993 ▸; Naismith & Sprang, 1998 ▸). Each CRD is cross-linked by three internal disulfides following the pattern AABCBC. The superposition of CD27 on the structure of TNFR1 (PDB entry 1ext; Naismith *et al.*, 1996 ▸) gives an r.m.s.d. of 1.0 Å for 74 common C^α^ atoms of the first two CRDs. While the core of the two structures is well preserved, the U-turns of the loops deviate by 3–4 Å, particularly where deletions in CD27 with respect to TNFR1 occur (Fig. 1 ▸ *a*). The different conformation of the N-terminus is likely to be owing to the Fab binding in the present structure.

CRD3 in CD27 is located much closer to CRD2 when compared with TNFR1 (Fig. 1 ▸ *a*). Superposition of the third CRDs of CD27 and TNFR1 requires a rotation of 36°. Such a variation in the relative orientation of CRD3–4 with respect to CRD1–2 has been noted previously upon comparison of the structures of different members of the TNFR superfamily or of independent copies of the same structure (Naismith *et al.*, 1996 ▸). The maximum difference of 49° was observed for the ligand-bound *versus* unbound forms of RANK (Liu *et al.*, 2010 ▸). The conserved C*X*C motif at the junction of CRD2 and CRD3 is thought to be the hinge that allows the CRDs to position themselves onto the binding regions of the ligands (Mongkolsapaya *et al.*, 1999 ▸). The corresponding residues in CD27 are Cys84-Ala85-Cys86.

Based on the structural similarity to the members of the TNFR superfamily, it is possible to identify a putative ligand-binding site in the CD27 structure. Superposition of CD27 on the structure of CD40 in complex with CD40L (PDB entry 3qd6; An *et al.*, 2011 ▸) indicates that the CD70 epitope is likely to include residues 57–68 of CRD2 (Fig. 1 ▸ *b*). Importantly, this is the region that shares 100% conservation between human and mouse CD27, whereas the sequence identity within residues 1–101 is 79% and the overall identity in the ECD is only 62%. This agrees with the observation that human CD70 can bind mouse CD27 and *vice versa* (Tesselaar *et al.*, 1997 ▸).

CD27 differs from the other members of the superfamily in two respects. The disulfide cross-linked N-terminal portion of the ECD (residues 1–101) is the shortest among the family members. The following 70 residues may be folded without disulfides or, more likely, do not form any tertiary structure. Another distinction of CD27 is the presence of a free cysteine at position 165, six residues before the transmembrane domain. Resting T cells express CD27 as a disulfide-linked homodimer (Camerini *et al.*, 1991 ▸), and one would expect that it is Cys165 that forms the cross-link. If so, the lengthy unstructured portion of the ECD may provide the necessary flexibility for the molecule, so that each of the two chains can bind a ligand. Moreover, both chains of the CD27 dimer could bind to the same trimer of CD70, leaving only one vacancy for another CD27 chain. This scenario is in accord with the fact that sCD70 is unable to trigger CD27-associated signaling pathways without additional cross-linking (Wyzgol *et al.*, 2009 ▸).

The membrane-proximal portion of the ECD contains two proline-rich motifs P*X*P*X*P*X*. One of them immediately follows CRD3 and spans residues 102–107 (PLPNPS), and the other spans residues 118–123 (PHPQPT). Proline-rich motifs mediate intermolecular interactions in many facets of the immune response, including antigen recognition, cell–cell communication and signaling (Freund *et al.*, 2008 ▸). Numerous adapter proteins involved in lymphocyte activation possess specific protein domains, such as the SH3 and WW domains, that selectively recognize proline-rich regions in their partners (Macias *et al.*, 2002 ▸). The interactions usually involve hydrophobic residues and mediate transient protein–protein interactions of low affinity. It is plausible to assume that CD27 may also have some partners involved in modulating its costimulatory signaling and that the proline-rich motifs represent their binding epitopes.

A number of mutations in the *CD27* gene have been identified that lead to the disruption of CD27–CD70 inter­action and impair immunity (van Montfrans *et al.*, 2012 ▸; Alkhairy *et al.*, 2015 ▸). Four of them, C33Y, R58W, C76Y and R87C (numbering of the mature CD27), map to the ECD. The mutations that either replace or introduce cysteine residues are apparently detrimental to the integrity of the structure, which is dependent on the correct disulfide cross-linking. The result would be a misfolded inactive protein. In this regard, the R58W mutation is seemingly neutral as it does not involve disulfides. This residue is unlikely to play a major structural role. It is located in the first loop of CRD2 cyclized by disulfide Cys45–Cys61 and does not make contacts with other structural elements of either this or another CRD. Its replacement by Trp should be accommodated without problem since Arg58 is completely exposed. The only sensible explanation of the pathological effect of this mutation is that it disrupts the interaction with CD70. Indeed, Arg58 falls exactly in the region of the postulated CD70-binding site and in essence supports the epitope hypothesis (Fig. 1 ▸ *b*).

### 3.2. CD27–Fab 2177 complex   

**Figure 2:**
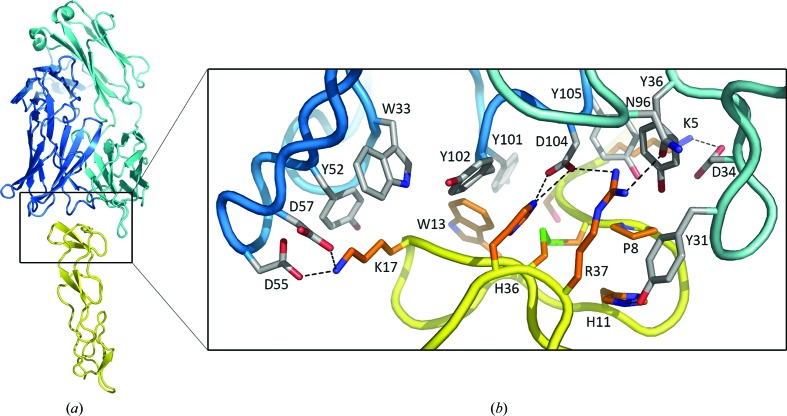
Interactions between CD27 and the Fab in the complex. (*a*) Ribbon representation of the structure, with CD27 in yellow, the Fab heavy chain in blue and the Fab light chain in cyan. (*b*) A close-up view of the boxed area. Side chains are shown as sticks and hydrogen bonds are shown by dashed lines.

The crystal structure of the CD27–Fab 2177 complex reveals the binding mode of the antibody (Fig. 2 ▸ *a*). Fab 2177 binds CD27 on top of the molecule at the site distal from the cell surface. The solvent-accessible surface area buried in the antibody–antigen interface is about 700 Å^2^ on each molecule. The shape-complementarity index is 0.80. The mAb epitope on CD27 is conformational and includes residues 5–17 and 36–37 from two stretches of the protein chain (the epitope is defined as the antigen residues within 4 Å of the antibody residues). In the center of the epitope are His36 and Arg37, which stack against two tyrosine residues of the antibody: Tyr31(L) from the light chain and Tyr102(H) from the heavy chain (Fig. 2 ▸ *b*). In addition to these interactions, His36 and Arg37 form hydrogen bonds to Asp104(H) of CDR H3 and Asn96(L) of CDR L3. Two lysine residues at the periphery of the epitope, Lys5 and Lys17, form salt bridges to Asp34(L), Asp55(H) and Asp57(H) and are engaged in stacking interactions with Tyr105(H) and Tyr52(H) through their aliphatic chains (Fig. 2 ▸ *b*).

A total of five residues from the light chain and nine residues from the heavy chain are in direct contact with CD27. All six CDRs are involved in antigen recognition, which is somewhat surprising given the relatively small paratope area. This is likely to be owing to the shape of the interface, where the long CDR L1 creates a concave antigen-binding surface of the mAb that embraces the convex surface of CD27 (Fig. 2 ▸ *a*). Compared with the structure of the same Fab in the ternary complex of CD27 with two noncompeting antibodies (PDB entry 5tlk; Obmolova *et al.*, 2017 ▸), no significant differences are observed. The r.m.s.d. for 231 C^α^ atoms of the variable domains is 0.39 Å. The CDR conformation is even more conserved, with an r.m.s.d. of 0.28 Å for 62 C^α^ atoms or 0.36 Å for all 486 non-H atoms.

The epitope of mAb 2177 is remote from the putative binding site of CD70, suggesting that both molecules may bind CD27 simultaneously. Indeed, mAb 2177 does not block the binding of the soluble form of the ligand, sCD70 (Obmolova *et al.*, 2017 ▸). It does however neutralize the cell-bound form of CD70, most likely by steric interference owing to its placement on top of the receptor chain. The orientation of Fab inline with the receptor axis is unique among anti-TNFR superfamily antibodies that have been structurally characterized. Typically, they bind around CRD1 and CRD2 on the side of the receptor molecule (Fellouse *et al.*, 2005 ▸; Li *et al.*, 2006 ▸; Adams *et al.*, 2008 ▸; Chodorge *et al.*, 2012 ▸; Graves *et al.*, 2014 ▸; Tamada *et al.*, 2015 ▸; Yamniuk *et al.*, 2016 ▸). The location of the epitope at the top edge provides an easier access for an antibody, which may be beneficial, particularly in the tumor microenvironment. For this reason, mAb 2177 may be considered as a potential cross-linking agent in the presence of CD70.

TNFR superfamily members are typically activated by ligand-induced trimerization or higher order oligomerization, resulting in initiation of intracellular signaling processes (Wajant, 2015 ▸). On the other hand, some of them can be activated by divalent mAbs even in the absence of their ligands or other cross-linking agents, such as secondary antibodies or Fcγ receptors. One explanation of mAb agonism postulates the existence of the pre-ligand assembly domains (PLADs) within receptors that mediate clustering in the absence of ligands (Chan *et al.*, 2000 ▸). No PLAD was observed in CD27; however, this receptor exists as an atypical disulfide-linked dimer and therefore seems to be predisposed to higher order clustering. In spite of this, all reported anti-CD27 mAbs, including mAb 2177, are non-agonistic per se (Wajant, 2016 ▸), suggesting that CD27 tetramerization is not sufficient for activation, and probably more receptor chains should cluster together to elicit signaling. Whether the epitope location plays any role in receptor activation remains to be seen.

## Supplementary Material

PDB reference: complex between human CD27 and antibody mAb 2177, 5tl5

## Figures and Tables

**Table 1:** Crystal data, X-ray data and refinement statistics Values in parentheses are for the highest resolution shell.

Crystal data	
Space group	*C*2
Unit-cell parameters (Å, °)	*a* = 274.20, *b* = 37.41, *c* = 61.08, α = 90, β = 98.82, γ = 90
Molecules per asymmetric unit	1 complex
*V*_M_ (Å^3^ Da^−1^)	2.58
Solvent content (%)	52
X-ray data	
Resolution (Å)	30–1.8 (1.85–1.80)
No. of measured reflections	159181 (11488)
No. of unique reflections	55970 (4054)
Completeness (%)	97.2 (96.5)
Multiplicity	2.8 (2.8)
*R*_merge_(*I*)	0.030 (0.252)
Mean *I*/σ(*I*)	20.0 (4.1)
*B* factor from Wilson plot (Å^2^)	33.8
Refinement	
Resolution (Å)	15.0–1.8
Completeness (%)	95.2
No. of reflections, working set	54736
No. of reflections, test set	1138
*R*_cryst_	0.197
*R*_free_	0.232
Total No. of atoms	4402
No. of water molecules	305
R.m.s.d.	
Bond lengths (Å)	0.008
Bond angles (°)	1.1
Mean *B* factor from model (Å^2^)	38.2
Ramachandran plot, most favored (%)	89.5
Ramachandran plot, disallowed (%)	0.2
